# Experience and perspectives of infection prevention staff of the COVID-19 response in Australian hospitals

**DOI:** 10.1186/s13756-022-01116-9

**Published:** 2022-06-02

**Authors:** Alisha Baswa, Philip L. Russo, Joseph S. Doyle, Darshini Ayton, Andrew J. Stewardson

**Affiliations:** 1 Department of Infectious Diseases, The Alfred Hospital and Central Clinical School, Monash University, Melbourne, Australia; 2 Department of Nursing Research, Cabrini Institute, Malvern, Australia; 3 Department of Nursing and Midwifery, Monash University, Frankston, Australia; 4 Department of Epidemiology and Preventive Medicine, Monash University, 553St Kilda Road, Melbourne, Australia

**Keywords:** Infection prevention and control, Australian Hospitals, COVID-19 OR coronavirus

## Abstract

**Background:**

Hospital infection prevention and control (IPC) staff have played a key role in adapting and implementing jurisdictional COVID-19 policy during the current pandemic. We aimed to describe the experiences of IPC staff in Australian hospitals during the COVID-19 pandemic to inform future pandemic preparedness plans.

**Methods:**

A cross-sectional study involving an online survey distributed to IPC practitioners employed in Australian hospitals. Survey content was informed by in-depth interviews, and addressed work conditions, redeployed workforce, personal protective equipment, communication, and guidelines. Participants were recruited through the mailing lists of Australasian College of Infection Prevention and Control and the Australasian Society of Infectious Diseases.

**Results:**

We received fully or partially completed responses from 160 participants, including 38 (24%) and 122 (76%) with nursing and medical backgrounds, respectively. Respondents reported access to sufficient information about PPE (75%, 114/152), PPE was of sufficient quantity (77%, 117/152) and was of sufficient quality (70%, 106/152). Barriers to infection prevention guideline implementation included frequently changing guidelines (57%, 84/148), timing of updates (65%, 96/148) and contradictory sources of information (64%, 95/148). Respondents described a need for better communication channels from government authorities to hospital IPC teams. All respondents described an increase in workload leading to difficulty completing work (63%, 97/154) and feeling burnt out (48%, 74/154).

**Conclusions:**

These data identify avoidable barriers to implementation of COVID-19 infection prevention guidance in Australian hospitals. These findings can inform future national preparedness strategies.

**Supplementary Information:**

The online version contains supplementary material available at 10.1186/s13756-022-01116-9.

## Background

Hospital infection prevention and control (IPC) teams have had a critical role in the COVID-19 response. In Australian hospitals, IPC units most frequently include infection control professionals, who are generally nurses who have undergone further training to specialise in this area, and infectious disease physicians [1]. This team of professionals are responsible for the health service infection control and prevention program which includes implementation and evaluation of IPC guidelines, education, surveillance, outbreak management and ensuring staff health [2].

We aimed to describe the experience of infection control professionals and infectious diseases physicians working in infection prevention and control (IPC) units in Australian hospitals during the COVID-19 pandemic. We aimed to capture the barriers and enablers of the response to the pandemic and develop recommendations that inform an ideal response in the future.

## Methods

We conducted an online cross-sectional survey of staff working in IPC teams in Australian hospitals.

### Setting

Australia is a federation of eight jurisdictions: six states and two territories. Inpatient healthcare is provided by public and private hospitals. During the COVID-19 pandemic, national infection prevention guidance related to COVID-19 was developed by the Infection Control Expert Group (ICEG), but hospital infection prevent teams (particularly public hospitals) generally refer to guidelines and directions from their own jurisdiction.

### Survey development

To inform the development of the survey, we conducted a literature review focussing on implementation of infection control policy in the context of an epidemic and performed semi-structured interviews with four infection control professionals from one hospital about their experiences of IPC during COVID-19. The main topic areas identified were; workload, workforce (redeployment of staff into IPC roles), personal protective equipment, communication, guidelines, education and training and outbreak response. We use the term ‘guideline’ to refer any written document that provides instruction about how infection prevention and control activities must or should be conducted within a hospital. This encompasses both recommendations and legally binding directives.

The survey was built using the electronic survey tool Qualtrics (Qualtrics, Provo, USA). It contained 8 domains with a total of 40 questions (Additional file 1). The majority of questions were in matrix format. All questions were mandatory with an option of ‘I don’t know’ or ‘Not applicable’. The survey was piloted by members of the research team and an external infection control professional.

We conducted this survey in accordance with the CHERRIES checklist [3].

### Recruitment

Participants were recruited through electronic mailing lists of the relevant professional societies; the Australasian College of Infection Prevention and Control (ACIPC) on the 10 August 2020 and Australasian Society of Infectious Diseases (ASID) on the 11 August 2020. Reminder emails were sent to both groups two weeks after the initial email to increase survey uptake. The survey was then closed on the 12 September 2020. At the time, both ACIPC and ASID had more than 1000 members, but we are unable to identify the total number of unique eligible individuals who were contacted: some individuals will be members of both societies and not all members would be eligible (in particular because not all will be involved in infection prevention in Australian hospitals).

### Data analysis

The survey was analysed using Stata version 16 (Statacorp, USA) for descriptive statistics and the free text responses were analysed using content analysis in Microsoft Excel (Version 16.40, 2019 Microsoft Corporation, USA).

### Ethics

Approval was granted by the Alfred Health Human Research Ethics Committee and was registered with the Monash University Human Research Ethics Committee.

## Results

We received 160 responses; 146 complete, 14 incomplete. Respondent characteristics are presented in Table 1. Respondents were predominantly infection control professionals (126/160, 76%), with greater than six years infection prevention experience (110/160, 69%) who were in a leadership role (116/160, 73%). The majority worked primarily in public metropolitan hospitals within IPC teams containing less than three full time equivalents. We received responses from all states and territories except the Australian Capital Territory, with Victoria and New South Wales being most frequently represented. 34/160 (21%) respondents described having experienced a COVID-19 outbreak in their hospital.

**Table 1:** Demographics of participants

	n	%
*Profession*		
Physician	36	23
Physician trainee	2	1
Infection control professional	122	76
*Years worked in infection prevention*		
< 1 year	7	4
1–5	43	27
6–10 years	43	27
11–15 years	30	19
16 years or more	37	23
*Leadership role*		
Yes	116	73
*Full time equivalent (FTE) infection prevention staff*		
< 1	37	23
1 or 2	55	34
3 or 4	32	20
≥ 5	29	18
Missing	7	4
*The hospital I primarily work in is located in the following area*		
Metropolitan	98	61
Regional	32	20
Rural	30	19
*The hospital I primarily work in is*		
Public	124	78
Private	32	20
I spend an equal time in both private and public hospitals	4	3
*The hospital I primarily work in is located in the following state/territory*		
VIC	55	34
NSW	53	33
QLD	17	11
NT	4	3
WA	19	12
SA	6	4
TAS	6	4
*Approximate number of beds at hospital*		
< 200	59	37
200–400	41	26
> 400	60	38
*Approximately number of confirmed COVID-19 inpatients treated at the hospital*		
0	51	32
1–10	42	26
11–25	20	13
> 25	43	27
I don’t know	4	3
*Outbreaks or clusters at the hospital*		
Yes	34	21
No	110	69
I don’t know	2	1
Missing	14	9

The survey covered the key domains of personal protective equipment (PPE), guidelines, communication, redeployment, training and personal experience.

### Personal protective equipment

**Fig. 1:**
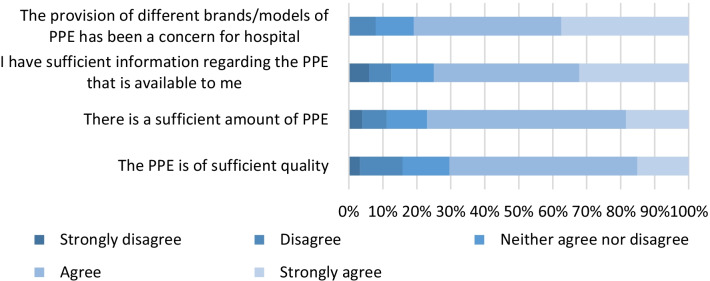
Challenges with PPE. N = 152 (8 survey responses missing)

In general, respondents either ‘agreed’ or ‘strongly agreed’ that they had sufficient information regarding PPE (123/152, 81%), and that PPE was available in both sufficient quantity (117/152, 77%) and quality (106/152, 70%) (Fig. 1). There was, however, general agreement that the provision of multiple different brands and models of PPE was the source of concern (Fig. 1).

### Guidelines

**Fig. 2:**
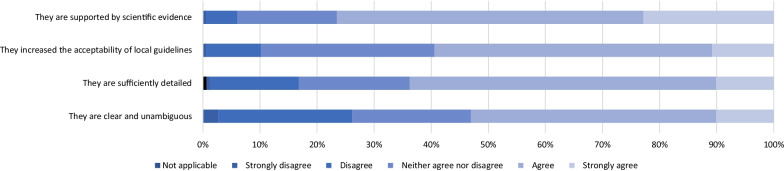
Infection prevention's opinion of government guidelines. N = 148 (12 survey responses missing)

More than half of respondents agreed or strongly agreed that government guidelines were supported by scientific evidence, they increased the acceptability of local guidelines, they were sufficiently detailed and were clear and unambiguous (Fig. 2).

**Fig. 3:**
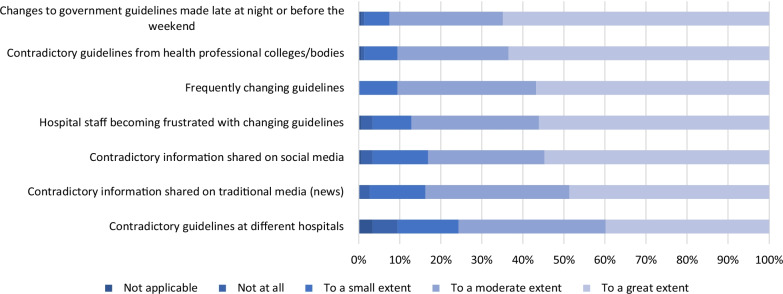
Factors impacting ability to implement guidelines. N = 148 (12 survey responses missing)

In contrast to general support for the *content* of government guidelines, respondents agreed with a number of barriers to their implementation (Fig. 3). Among such barriers were the high frequency of guideline modification (84/148, 57%), the release of guideline updates late at night or before the weekend (96/148, 65%), and contradictory information from professional societies (95/148, 64%), other hospitals (59/148, 40%), news media (73/148, 49%), and social media (81/148 55%) (Fig. 3).

### Communication

**Fig. 4:**
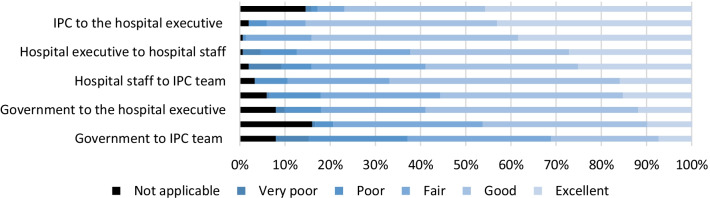
Communication channels for infection prevention information. N = 151 (9 survey responses missing)

The four stakeholder groups identified were government, hospital executive, hospital IPC and hospital staff. The channels that infection prevention thought that had an excellent flow of information was within the IPC team and IPC to hospital executive. The communication channel that infection prevention thought was the worst was the government to IPC (Fig. 4).

### Redeployment

**Fig. 5:**
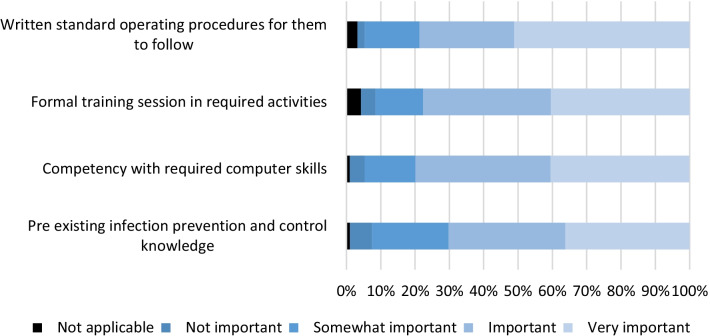
training necessary for successful redeployment of healthcare workers to infection prevention. N = 92

Ninety-two respondents indicated that redeployment occurred at their hospital. The respondents identified written standard operating procedures, formal training, and competency with computing skills as being more important for successful redeployment of the IPC team than pre-existing IPC knowledge (Fig. 5).

### Personal experience

**Fig. 6:**
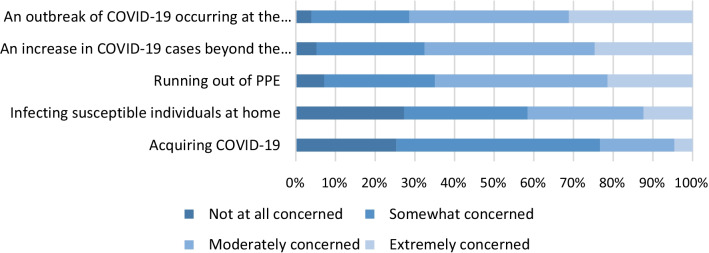
Concerns of infection prevention working in a hospital. N = 154 (6 survey responses missing)

**Fig. 7:**
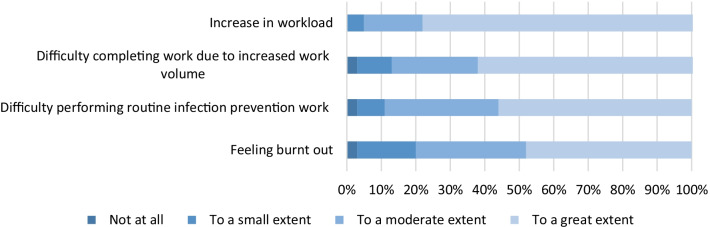
Experience of workload for infection prevention. N = 154 (6 survey responses missing)

The greatest concern for respondents was an outbreak occurring at the hospital they worked in and least concerned about acquiring COVID-19 themselves (Fig. 6). All respondents reported an increase in workload (Fig. 7). The majority of respondents found that the increased workload prevented from completing work (97/154 63%) and difficulty in completing routine infection prevention work (86/154 56%). This then led to feelings of burn out to a great extent in respondents (74/154 48%).

## Discussion

This is the first Australian study to explore the experience of IPC teams in response to the COVID-19 pandemic. Hospital infection control policies are part of a system of integrated policies and practices that aims to control disease transmission, as was seen in the response to COVID-19 in many countries. [4–6] This study identified through interviews and surveys key components of infection control implementation strategies that could be improved upon for future outbreaks, with a focus on the themes of PPE, guidelines, communication and redeployment strategies. We have synthesised the findings of this survey into a series of recommendations (Box 1).

**Box 1:** Summary of key recommendations

Domain	Recommendation(s)
Guidelines	One source of information through collaboration with colleges and professional societies Government guidelines released with enough time to implement
Staffing and deployment	Increase baseline staffing Process for selecting appropriate staff e.g. IT skills Processes for training redeployed staff e.g. written standard operating procedures
Communication methods	A clear well understood strategy for communication from government to infection prevention teams
PPE	Ensure standardised PPE are part of stockpile

*IT* information technology, *PPE* personal protective equipment

News outlets within Australia regularly commented that healthcare workers have inadequate access to PPE [7]. This is consistent with surveys of frontline healthcare workers in other countries [8, 9]. In contrast, our data suggests that hospital infection control teams believed that their hospitals had access to sufficient quantity and quality of PPE. This discrepancy may, to a large extent, be explained by the fact that while sufficient PPE was generally available in Australian hospitals to implement the guidelines as they were written (i.e. a reliance on surgical masks for care of COVID-19), there were prominent calls for increasing use of P2/N95 respirators by frontline healthcare workers [10]. A finding that was not reported during previous epidemics was that the provision of different brands and/or models of PPE caused concern in hospital staff. This emphasises how limiting the amount of changes within the constantly shifting environment of a pandemic can lessen concern for hospital staff.

Previous studies identified that a lack of adequate implementation of guidelines, constant guideline changes and alternative sources of information were challenges associated with guidelines [11–13]. We found that despite these learnings from previous epidemics, Australian hospitals continue to face similar challenges. In addition to this, our study identified that updates to government guidelines published out of hours when there were fewer IPC staff to adapt their own internal hospital guideline was also a barrier to implementation.

We explored the perceived quality of communication between four stakeholder groups involved with the COVID-19 infection prevention in hospitals: government, hospital executive, hospital IPC teams and hospital staff. While there was good communication within IPC teams and from IPC teams to hospital executive there was a perceived poor quality of communication from government to hospital executive and IPC teams. Lack of feedback routes from healthcare workers to policymakers was previously identified as an issue in the public inquiry conducted by Canada into the SARS epidemic [14].

This study identified that written standard operating procedures were the most useful tool for successful redeployment. So far there are no studies investigating the effect of redeployment during COVID-19. We also identified that respondents to this survey were more concerned about an outbreak occurring at their hospital rather than acquiring COVID-19 themselves. This could be because the respondents of the survey were within working age range and the majority of deaths within Australia have been reported in those 70 years old and above [15].

Given that this cohort will continue to be an integral part of the COVID-19 response, and responses to future pandemics, we suggest that these data should be used to inform future pandemic planning. This survey has national representation from different jurisdictions and types of hospitals. This study was conducted during the pandemic ensuring that there was no recency bias. Notwithstanding the uniqueness, this project has several limitations. First, is the small sample size relative to the total number of potentially eligible individuals, which renders the results vulnerable to selection bias. The participation rate may be explained by the fact that this survey was conducted during the pandemic, when IPC staff were time poor and might not have been available to complete the survey. Finally, these results—by design—only represent the perspective of IPC staff. Within the COVID-19 response there are many different stakeholders involved from frontline healthcare workers to operational managers within the hospital. While the recommendations expressed in these results might help IPC staff it might not be feasible to achieve due to constraints within different areas of the hospital.

## Conclusion

With this study, we have aimed to ensure that the lessons learnt during the course of the COVID-19 pandemic are not forgotten, but that they are instead leveraged for the benefit of our future pandemic preparedness.

## Supplementary Information

**Additional file 1.** Exploring the experience and perspectives of infection preventionin managing COVID-19 to inform future pandemic planning.

## Data Availability

All data generated or analysed during this study are included in this published article [and its Additional file 1].

## Abbreviations

ACIPC: Australasian Collection for Infection Prevention and Control
ASID: Australasian Society for Infectious Diseases
ICEG: Infection control expert group
IPC: Infection prevention and control
PPE: Personal protective equipment
