# Supplementary material for: Experience and perspectives of infection prevention staff of the COVID-19 response in Australian hospitals
Source: Antimicrob Resist Infect Control. 2022 Jun 2;11:77. doi: 10.1186/s13756-022-01116-9 (PMC9161183; doi:10.1186/s13756-022-01116-9)
Supplement: Supplementary file 1 — Additional file 1. Exploring the experience and perspectives of infection preventionin managing COVID-19 to inform future pandemic planning. [file 13756_2022_1116_MOESM1_ESM.pdf]

Introduction

Exploring the experience and perspectives of infection prevention in managing COVID-19 to inform future pandemic planning

**Principal Investigator**	Dr Andrew Stewardson
**Co-Investigators**	Ms Alisha Baswa, Dr Joseph Doyle, A/Prof Phil Russo, Dr Darshini Ayton
**Project Number**	303/20
**Project Sponsor**	Monash University
**Location**	Alfred Hospital

- You are invited to participate in this research project because you are employed in an Australian hospital and are either a member of:
    - Australian College of Infection Prevention and Control (ACIPC)
    - Healthcare Infection Control Special Interest Group (HICSIG) of the Australasian Sociey of Infectious Disease

- **Aim:** to determine the experiences of individuals working in an infection control unit during a pandemic.
- **Benefits:** the opportunity to inform future research and policies surrounding infection control implementation.
- **What will happen to survey data:** All information obtained from you will be anonymous. The data from this survey will be used for Alisha Baswa's honours project and for a publication.

- **Ethics:** The ethical aspects of this research project have been approved by the Alfred Hospital Ethics Committee. This project will be carried out according to the National Statement on Ethical Conduct in Human Research (2007) produced by the National Health and Medical Research Council of Australia.

Further questions: Please contact abas37@student.monash.edu
For complaints: If you have any complaints about any aspect of the project, the way it is being conducted or any questions about being a research participant in general, then you may contact:

Position	Complaints Officer, Office of Ethics & Research Governance, Alfred Health
Contact	(03) 9076 3619 OR research@alfred.org.au

Instructions:

The survey should take approximately 10 minutes to complete.

If you are unable to complete the survey in one sitting you will be able to save and complete it within 1 week.

The survey covers the following areas:

- Your role and information about your hospital
- Working during a pandemic
- Staffing and redeployment
- PPE
- Communication
- Guidelines
- Education and Training
- Leadership and Teamwork
- Outbreak and Response

If you agree to participate please press forward arrow button below.

Your Role and Information About Your Hospital

Your Role and Information About Your Hospital

What is your profession?

- ○ Physician
- ○ Physician trainee
- ○ Infection Control Professional
- ○ Other - please specify ______

How many years have you worked in infection prevention?

- ○ < 1 year
- ○ 1-5 years
- ○ 6-10 years
- ○ 11-15 years
- ○ 16 years or more

Are you in a leadership role in the infection control unit?

- ○ Yes
- ○ No
- ○ I don't know

Please answer the following questions based on the hospital in which you spend the majority of your time.

The hospital I primarily work in has approximately _____ beds.

- ○ <200
- ○ 200-400
- ○ >400

The hospital I primarily work in is located in the following state/territory:

The hospital I primarily work in is located in the following area:

- ○ Metropolitan
- ○ Regional
- ○ Rural
- ○ I don't know

The hospital I primarily work in is:

- ○ Public
- ○ Private
- ○ I spend equal time in both private and public hospitals

Approximately how many confirmed COVID-19 inpatients have been treated at the hospital?

- ○ 0
- ○ 1-10
- ○ 11-25
- ○ >25
- ○ I don't know

Working during a pandemic

Working During a Pandemic

With regard to COVID-19, which of the following tasks have you performed? Select all that apply.

- ☐ Contact tracing
- ☐ Managing staff furlough and return to work
- ☐ Hospital staff education and training (e.g. teaching staff about PPE)
- ☐ Developing and/or updating internal guidelines

- ☐ Responding to queries from hospital staff (by phone or email)
- ☐ COVID-19 swab collection
- ☐ Internal planning/strategy meetings
- ☐ Responding to media
- ☐ Training redeployed staff
- ☐ Other - please specify

To what extent have you experienced the following during the COVID-19 pandemic:

	Not at all	To a small extent	To a moderate extent	To a great extent
Increase in workload	○	○	○	○
Difficulty completing work due to increased work volume	○	○	○	○
Feeling burnt out	○	○	○	○
Difficulty performing routine infection prevention work	○	○	○	○

How concerned are you about the following:

	Not at all concerned	Slightly concerned	Moderately concerned	Extremely concerned
Acquiring COVID-19	○	○	○	○
An outbreak of COVID-19 occurring at your hospital	○	○	○	○
Infecting susceptible individuals at home e.g. elderly parents	○	○	○	○
Running out of PPE	○	○	○	○
An increase in COVID-19 cases beyond healthcare system capacity as seen overseas	○	○	○	○

Do you believe your hospital is currently better equipped to deal with a pandemic compared to the start of the year?

- Yes
- No - please specify why not [ ]
- I don't know

Do you think there has been an increase in public appreciation for your job since the pandemic?

- Not at all
- To a small extent
- To a moderate extent
- To a great extent

Staffing and Redeployment

Staffing and Redeployment

How many full time equivalent (FTE) worked in the infection prevention team before COVID-19?

- <1
- 1
- 2
- 3
- 4
- 5
- >5

During COVID-19, have staff been redeployed from other areas within the hospital to your infection prevention team?

- Yes
- No

Which of the following were important to successful redeployment:

	Not important	Somewhat important	Important	Very important	App
Pre exisiting infection prevention and control knowledge	○	○	○	○	
Competency with required computer skills	○	○	○	○	
Written standard operating procedures for them to follow	○	○	○	○	
Formal training session in required activities	○	○	○	○	

PPE

Personal Protective Equipment

To what extent do you agree with the following statements about PPE in the hospital you primarily work in:

	Strongly disagree	Disagree	Neither agree nor disagree	Agree	Strongly Agree	Ap
There is a sufficient amount of PPE	○	○	○	○	○	
The PPE is of sufficient quality	○	○	○	○	○	
I have sufficient information regarding the PPE that is available to me	○	○	○	○	○	
The provision of different brands/models of PPE has been a concern for hospital staff	○	○	○	○	○	

Communication

Communication

Rate the quality of the flow of information between the following groups during COVID-19:

(IPC: Infection Prevention and Control)

	Very Poor	Poor	Fair	Good	Excellent	Not applicable
Government to the hospital executive	○	○	○	○	○	○

	Very Poor	Poor	Fair	Good	Excellent	Not applicable
Government to IPC team	○	○	○	○	○	○
Hospital executive to government	○	○	○	○	○	○
Hospital executive to IPC team	○	○	○	○	○	○
Hospital executive to hospital staff	○	○	○	○	○	○
IPC to the hospital executive	○	○	○	○	○	○
IPC to IPC (within the team)	○	○	○	○	○	○
IPC to hospital staff	○	○	○	○	○	○
Hospital staff to hospital executive	○	○	○	○	○	○
Hospital staff to IPC team	○	○	○	○	○	○

Rate the quality of the following methods of communicating infection prevention guidance to hospital staff during the COVID-19 pandemic:

(IPC: Infection Prevention and Control)

	Very poor	Poor	Fair	Good	Excellent	Not applicable
Email	○	○	○	○	○	○
Intranet	○	○	○	○	○	○
Online staff forum	○	○	○	○	○	○
Online teaching resources	○	○	○	○	○	○
Ad hoc discussion on the wards	○	○	○	○	○	○
Providing information during clinical meetings	○	○	○	○	○	○

Guidelines

Infection Prevention Guidelines

To what extent did the following impact your ability to implement guidelines:

	Not at all	To a small extent	To moderate extent	To a great extent	Not applicable
Frequently changing guidelines	○	○	○	○	○
Changes to government guidelines made late at night or before the weekend	○	○	○	○	○
Hospital staff becoming frustrated with changing guidelines	○	○	○	○	○
Contradictory guidelines from health professional colleges/bodies	○	○	○	○	○
Contradictory guidelines at different hospitals	○	○	○	○	○
Contradictory information shared on social media	○	○	○	○	○
Contradictory information shared on traditional media (news)	○	○	○	○	○

To what extent do you agree with the following statements about COVID-19 infection guidelines provided by your local state/territory government:

	Strongly disagree	Disagree	Neither agree nor disagree	Agree	Strongly Agree	Not Applicable
They are supported by scientific evidence	○	○	○	○	○	○
They are clear and unambiguous	○	○	○	○	○	○
They are sufficiently detailed	○	○	○	○	○	○
They increased the acceptability of local guidelines	○	○	○	○	○	○

Education and Training

Education and Training

Rate how useful the following strategies were for training staff on correct use of PPE:

(IPC: Infection Prevention and Control)

	Not useful	Slightly useful	Moderately useful	Extremely useful	Not applicable

	Not useful	Slightly useful	Moderately useful	Extremely useful	Not applicable
Demonstration of PPE	○	○	○	○	○
Formal education on COVID-19 e.g. how it's transmitted	○	○	○	○	○
Audit and feedback on the ward by IPC staff	○	○	○	○	○
Utilising nurse educators	○	○	○	○	○

Leadership + teamwork

Leadership and Teamwork

Rate the quality of the following in the hospital your primarily work at:

	Very Poor	Poor	Fair	Good	Excellent	Not Applicable
Leadership provided by hospital executive	○	○	○	○	○	○
Team work within your unit	○	○	○	○	○	○
Safety culture on the wards	○	○	○	○	○	○

Outbreak response

Outbreak Response

Have you had any outbreaks or clusters at the hospital?

- ○ Yes
- ○ No
- ○ I don't know

Do you have enough people to perform contact tracing?

- ○ Yes
- ○ No

○ I don't know

Were any wards closed in response to the outbreak?

○ Yes
○ No
○ I don't know

Future Recommendations

Future Recommendations

What recommendations would you make for the future pandemics and infection control? (optional)

Powered by Qualtrics
